# Location estimation of approaching objects is modulated by the observer’s inherent and momentary action capabilities

**DOI:** 10.1007/s00221-016-4637-1

**Published:** 2016-04-27

**Authors:** Manasa Kandula, Dennis Hofman, H. Chris Dijkerman

**Affiliations:** Helmholtz Institute, Experimental Psychology, Utrecht University, Heidelberglaan 1, 3584 CS Utrecht, The Netherlands

**Keywords:** Location estimation, Action capability, Perception, Embodied perception, Affordances

## Abstract

Action capability may be one of the factors that can influence our percept of the world. A distinction can be made between momentary action capability (action capability at that particular moment) and inherent action capability (representing a stable action capability). In the current study, we investigated whether there was a biasing effect of these two forms of action capability on visual perception of location. In a virtual reality room, subjects had to stop a moving ball from hitting a pillar. On some trials, the ball disappeared automatically during its motion. Subjects had to estimate the location of the ball’s disappearance in these trials. We expected that if action is necessary but action capability (inherent or momentary) is limiting performance, the location of approaching objects with respect to the observer is underestimated. By judging the objects to be nearer than they really are, the need to select and execute the appropriate action increases, thereby facilitating quick action (Cole et al. in Psychol Sci 24(1):34–40,  2013. doi:10.1177/0956797612446953). As a manipulation of inherent action capability in a virtual environment, two groups of participants (video game players vs. non-video game players) were entered into the study (high and low action capability). Momentary action capability was manipulated by using two difficulty levels in the experiment (*Easy* vs. *Difficult*). Results indicated that inherent and momentary action capabilities interacted together to influence online location judgments: Non-players underestimated locations when the task was *Difficult*. Taken together, our data suggest that both inherent and momentary action capabilities influence location judgments.

## Introduction

In order to be able to interact effectively with the objects in our nearby surrounding, we have to be able to first gauge how far they are from us. When interacting with the object, it is important to represent object properties in relation to our own action capabilities. Indeed, currently a large amount of evidence is available that suggests that our judgements of the external world and the objects in it are biased towards our own action capabilities (Proffitt et al. 2003; Witt et al. 2005).

Evidence for such a mechanism comes from the research on affordances. According to this idea, initially proposed by Gibson (1978), the utilities of an object, or *affordances*, are subject to constraints imposed by the action repertoire of the user. The term affordances, however, is an umbrella concept that covers a vast range of concepts that look at the influence of an observer’s ability to interact with an object on his perceptual judgements about the same object. The current experiment attempts to shed new light on action capability-based modulation of perceptual judgements in the context of object location judgements.

The judgements of the space around us have been demonstrated extensively as not being veridical. For instance, Witt et al. (2005) have demonstrated that our perception of the distance of an object is influenced by our intention and ability to interact with it. In this experiment, participants had to estimate the distances of targets that are ordinarily out of reach while holding a tool. Participants underestimated the distances of the targets when they were asked to reach for the object with the tool, and not when they were just passively holding the tool. Also, when they were asked to reach for the object without the tool, these underestimations did not occur. In another study, Proffitt et al. (2003) demonstrated that hills were judged as steeper when subjects were asked to carry a heavy load. Similarly, objects have been judged as closer when they were approaching the person in comparison with when they were receding from him (Takahashi et al. 2013). A recent study that could provide a framework for the interpretation of these results is the 2013 study by Cole et al. (2013) that showed that threatening objects were perceived to be closer than they really were. They propounded the *threat*-*signal hypothesis* which states that perceiving the threatening object as closer than it really is would encourage the observer to take quick action to withdraw from it. That is, misjudged proximity is argued to promote urgent action. Overall, these accounts show evidence to support the motivational nature of our perception: that is our current goals and actions influence how we perceive the external world.

The effects of either inherent action capability (Taylor et al. 2011; Witt et al. 2009) or momentary action capability (Proffitt et al. 1995, 2003; Schnall et al. 2010) on perceptual biases have also been studied extensively. According to the Witt (2011), *inherent* action capability refers to the set of motor skills and strategies the person possesses to perform the task effectively. In addition, *momentary* action capability is defined as the robustness of these skills to cope with the current task difficulty level. To our knowledge, however, few studies have directly compared the effects of both forms of action potential in the cognitive domain. One exception is the study by Witt et al. (2008), who compared the effects of golfing performance on judged hole size and found that people who performed badly, judged the hole as being smaller (negative correlation between performance and perceived size) while their golf handicap (measure of general ability) did not correlate. They concluded that the current performance influenced perceptual judgements while general ability did not. However, all their subjects were relatively unskilled golfers, making it *Difficult* to predict whether seasoned golfers would be better at estimating hole size. That is, the design used was unable to provide a large enough range in inherent performance abilities to be able to separate the effects of general ability and current performance on perception.

We therefore aimed to investigate the effects of both inherent and momentary action capabilities on the location estimation of approaching objects in the same task. Our task was performed in a first person virtual reality setting. The goal of the subject was twofold. The first was to stop a moving ball before it hit a pillar standing next to the subject. The next task was to estimate the location at which the ball disappeared. We expect that both forms of action capability interact to produce the final perceptual estimate of location. Inherent action capability is compared by testing two groups of people: one group is expected to possess quick responses (video game players, VGPs) and the other is expected to possess average responses (non-video game players, NVGPs). Momentary action capability is manipulated for each individual by introducing two levels of task difficulty in stopping the moving ball.

VGPs were selected as the high inherent action capability group for this study as they are regularly exposed situations where quick decisions need to be made and appropriate actions need to be selected and executed quickly. Given the general fast pacing in these games, video games expose people to unpredictable and rapidly changing situations. There is currently a large body of research that shows that video game players possess enhanced visual, perceptual and cognitive abilities (Achtman et al. 2008; Cardoso-Leite and Bavelier 2014). More specifically, video game players have been shown to possess better motor coordination (Borecki et al. 2013), and faster reaction times (Castel et al. 2005) than non-players.

## Materials and methods

### Participants

Participants were recruited by means of digital advertisements and flyers. Persons between the age of 18 and 35 were included, if they indicated they had no mental health problems and did not suffer from visual problems other than sight correction, nor had trouble with stereopsis. Participants were unaware of the objective of the study and received credit points or financial remuneration. If responders indicated that they played video games more than 3 h a week, they were assigned *video game player* group (VGP). Responders who played video games <3 h a year, or never played video games, were assigned to the *non-video game player* (NVGP) group.

### Design

**Fig. 1:**
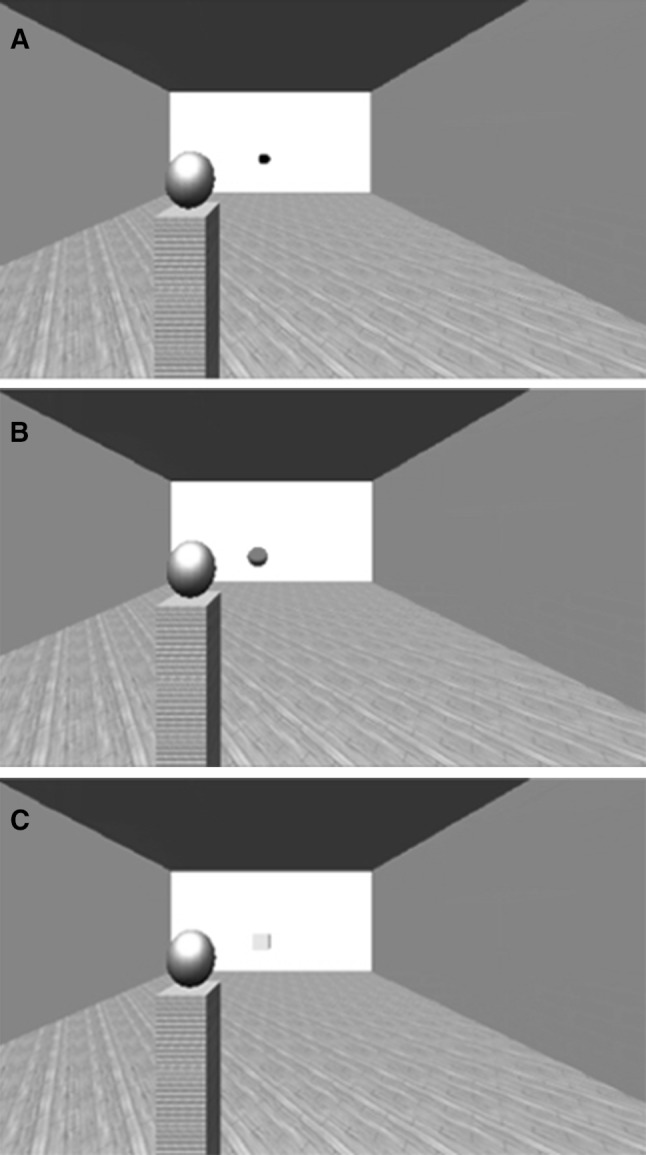
2-Dimensional recreations of the virtual room projected onto the right eye of the participant. **a** The ball is currently *black* in colour and approaching the pillar. **b** This is a Response type trial. The ball has turned *red*, indicating that the subject may now stop the ball. **c** The location estimation cube. This cube can be moved by the subject along the trajectory of the ball to indicate where the ball disappeared (colour figure online)

In order to investigate the relationship between action capabilities and location perception, participants were asked to estimate the location of an approaching object in a virtual environment, viewed from the first person perspective. Participants were viewing an empty room with a pillar standing on their left side, close to them. The task was to stop a moving ball from hitting this pillar. Also, subjects had to estimate the location at which the ball disappeared. There were two types of trials in the experiment: *Response* trials and *Estimation* trials. These two trial types were included to gather response time information and location estimation information independently of one another. In the *Response* trial, the approaching object was a black ball that moved towards the pillar (Fig. 1a). The ball started at a certain (the details are provided in the *procedure* section) distance, and the task of the subject was to stop the ball before it hit the pillar. During its motion, the ball would turn red in colour (Fig. 1b). Participants could stop the ball only after it turned red, by pressing the space bar. After the space bar was pressed, the ball disappeared. If the ball was not stopped on time (before it hit the pillar), an aversive sound would be played through the headphones. The dependent variable (DV) of interest in these trials was the response time (RT: the time at which they first pressed the space bar to stop the ball). In addition to this, participants were also instructed to estimate the location at which the ball turned red. These estimations were not our main variables of interest and were included only to encourage the participant to pay attention to the location at all times.

*Estimation* trials were included to obtain location estimation (*LE*) information that was uncorrupted by the necessity to stop the ball. In these trials, the approaching object was again a black ball that moved towards the pillar (Fig. 1a). Instead of turning red (as in the case of *Response* trials), the ball disappeared automatically during its motion. The participants had to indicate the location at which the ball disappeared. Until the ball either turned red, or automatically disappeared, the participant was not aware of whether the trial was going to be an *Estimation* trial or a *Response* trial. By doing this, we expected that the level of urgency induced in that block would also affect the perceived locations in the *Estimation* trials. The ball could disappear (Estimation trials) or turn red (Response trials) at one of the two possible distances: 30 units (*Near* condition) and 50 units (*Far* condition) from the subject. Each distance unit used in the virtual world was approximately equal to 10 centimetres.

Location estimations were given by moving a cube (Fig. 1c), that was the same size as the ball, towards the location in space that the ball occupied when it either turned red (in the Response trial) or automatically disappeared (in the Estimation trial). The cube could be moved along the same trajectory that the ball travelled.

In order to be able to test the effects of the momentary action capability against the effects of inherent action capability, we added two difficulty levels in separate blocks. The *Easy* and *Difficult* blocks differed only in terms of the number of button presses required to bring the ball to a halt in the Response type trials. In the *Easy* block, one press of the space bar was sufficient to bring the ball to a halt. In the *Difficult* block, the space bar needed to press five times to bring the ball to a halt. Crucially, in the *Difficult* and *Easy* blocks, the embedded *Estimation* trials (trials where the location estimations were required) were identical. The order of the blocks was randomised for each participant.

In summary, the study comprised of two groups (VGP and NVGP). Each group performed two blocks of the task (*Easy* block and *Difficult* block). Each block consisted of two types of trials (Response trials and Estimation trials). The location at which the ball turned red (in Response trials) or disappeared automatically (in Estimation trials) could be at 30 units or 50 units from the subject (Near or Far). Each condition was repeated 10 times, yielding a total of 40 trials per difficulty block, and 80 trials in the experiment (excluding the practice trials).

### Materials

The experiment was conducted using an Oculus Rift DK1 virtual reality headset, which was connected to a PC running on Windows. Headphones (Sennheiser) were used to administer pre-programmed audio instructions. Participants could respond using the space bar and arrow keys on a keyboard. The space bar was used to stop the ball, and the up and down arrow keys were used to perform the location estimation. The experiment was programmed using the Unity 4.3.4 game engine.

### Procedure

The study was carried out in a controlled laboratory environment at Utrecht University. Firstly, participants were briefed in writing about the background of the study and what was expected from them. Next, they were asked to sign an informed consent form. All procedures were conducted in accordance with the guidelines dictated in the Declaration of Helsinki.

Participants were seated behind a desk, with their head resting on a chin rest. Prior to every block, participants received written instructions, during which they did not wear the head mounted display and headphones. All of the instructions emphasised that they had to estimate the location as precisely as possible and that they had to respond as fast as possible when the ball turned red. Next, the head mounted display and earphones were put on and a practice block was started. This was performed to acclimatise the participant to the virtual environment and to incorporate the appropriate sense of urgency necessary for that block. The practice block consisted of ten trials.

Pre-programmed auditory instructions about how to start the trial were given automatically before each trial through the headphones. All trials started with a fixation point at the back wall of the virtual room, at a location of 102 units from the view of the participant and a height of 10 units. Each distance unit used in the virtual world was approximately equal to 10 cm. The fixation dot was projected for a duration between 500 and 1500 ms. After it disappeared, a black ball started approaching from the opposite wall in a straight line. The starting location of the ball was randomised to be between of 90–100 units and at the ball always travelled at a height of 1.5 units below eye level. The size of the ball was 3 × 3 × 3 units, and it travelled at a speed of 20 units/s. At the left side, a pillar was located at 1 unit in front and 3.6 units left of the participant. The location where the ball turned red (*Response* trials) or disappeared (*Estimation* trials) was 30 (*near*) or 50 (*far*) units. It was possible to stop the ball after it turned red in the *Response* trials, by pressing the space bar.

After the ball had disappeared, pre-programmed instructions about how to indicate the location were given automatically through the headphones. Participants had to move a blue cube that appeared at the back of the virtual room (100 units away from them) to the estimated location, by pressing the up and down arrow keys. The cube moved over the same trajectory as the ball and had the same size (3 × 3 × 3 units).

Lastly, at the end of both blocks, participants were debriefed and were asked to indicate on a questionnaire how *Difficult* they felt the condition where multiple presses were required to stop the ball (*Difficult*) was compared to the condition where one button press was required to stop the ball (*Easy*). Their responses were given on a scale from 1 to 5 where 1 indicated *not Difficult* and 5 indicated *very Difficult*.

## Results

Out of the 32 responders, 13 were assigned as VGPs (9 males, 1 left handed, Mean Age = 21.92, SD = 3.04) and 12 NVGPs (3 males, Mean Age = 25.9, SD = 4.68) and 7 people who belonged to neither group were excluded from the analysis.

Analysis of the error rates (trials where the subjects failed to stop the ball on time) indicated that all subjects were able to stop the ball successfully and the rate of failure was <1 %.

### Location estimation

The analysis performed in this section used location estimates obtained from the *Estimation trials*, to prevent the influence of performing dual-task (stopping the ball and reporting the location in the *Response trials*) on the obtained location estimates. See the footnote for an overview of the results obtained from analysing the location estimates in the *Response* trials.^1^

In order to ensure that people were able to distinguish between the *Near* and *Far* distance conditions, the raw location estimates for the *Near* and *Far* distance conditions, for each block (*Difficult* and *Easy*) and group (VGP and NVGP) were entered into a paired sample *t* test. All tests yielded significant differences (*p* < 0.001, for all cases).

**Fig. 2:**
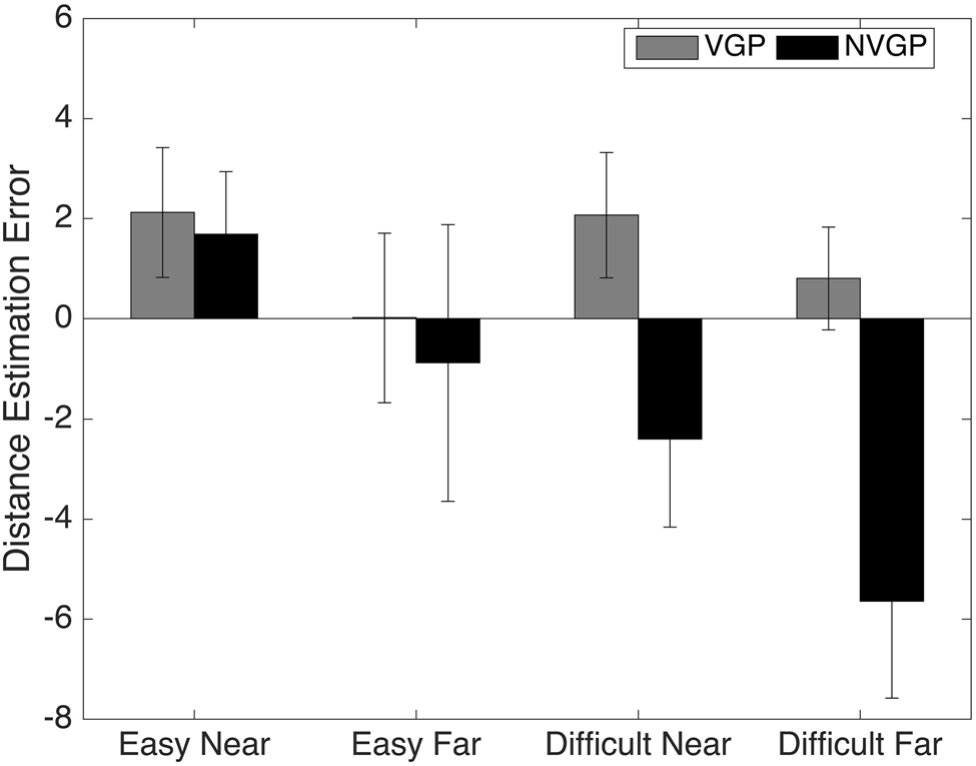
Location estimation error in distance units of the virtual world. Each distance unit is approximately equal to 10 cm. *Positive errors* were overestimations, where subjects estimated the ball to be further away than themselves. *Negative errors* were underestimations. The *error bars* represent the standard error, corrected for within-subject error

Next, the location estimation errors (LE) were calculated individually for each subject by subtracting the estimated location from the actual location. Negative errors indicated that subjects estimated the ball as being closer to them, and vice versa. Finally, the LE were averaged for all subjects, separately for each block and distance condition (Fig. 2).

In order to test the effects of group (NVGP, VGP), difficultly (*Easy*, *Difficult*) and distance (Near and Far) on the location estimates, we conducted a mixed between-group, within-subject design ANOVA. The within-subject factors were difficulty and distance. The dependant variable was the LE’s. The results showed a main effect of difficulty [*F*(23,1) = 5.093, *p* = 0.034, *n*^2^ = 0.181] and distance [*F*(23,1) = 11.395, *p* = 0.003, *n*^2^ = 0.331]. The factor difficulty also interacted with group [*F*(23,1) = 7.095, *p* = 0.014, *n*^2^ = 0.236]. No other factors were significant. Pairwise comparisons between *Easy* and *Difficult* conditions revealed that locations were underestimated significantly for the *Difficult* condition [*t*(24) = 2.25, *p* = 0.034]. Exploring the interaction of difficulty with group revealed that NVGPs underestimated the location of the ball in the *Difficult* condition. In order to test these effects, we conducted one sample *t* tests per difficulty and group to test which location estimation errors were significantly different from zero. The test revealed that only in the *Difficult* condition for the NVGP group were the LE significantly different [Near: *M* = − 2.39, SD = 3.34, *t*(11) = − 2.480, *p* = 0.031, Far: *M* = − 5.64, SD = 5.32, *t*(11) = − 3.674, *p* = 0.0043].

Overall, the location estimate data reveal that in the *Easy* condition, for either distance, both VGPs and NVGPs were accurate in estimating the location at which the ball disappeared. In the *Difficult* condition, the VGPs were again accurate in estimating the location at which the ball disappeared, while NVGPs underestimated the locations at both the distances.

### Response times

**Fig. 3:**
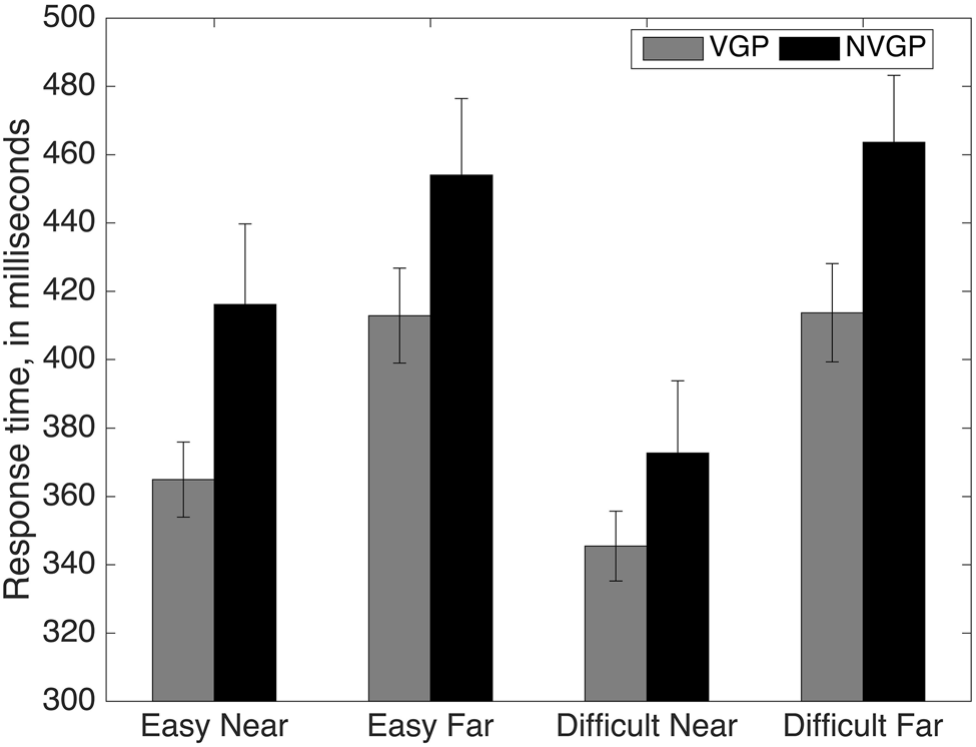
Response times to the stop signal. The *error bars* for each condition were created by calculating the confidence intervals (*α* = 0.05) after correcting for between-subject differences in the scores

Reaction times (RTs) were calculated for each subject by subtracting the time at which the subject pressed the stop button (for the first time in the *Difficult* condition) from the time at which the ball turned red. The RTs were averaged for each subject, separately for each block and distance condition (Fig. 3).

In order to test the effects of group (NVGP vs. VGP), difficulty (*Easy*, *Difficult*) and distance (Near and Far) on the response times, we conducted mixed between-group, within-subject design ANOVA. The within-subject factors were difficulty and distance. The results showed a main effect of group: VGPs were faster than NVGPs [*F*(23,1) = 5.59, *p* = 0.027, *n*^2^ = 0.196]. The factor distance [*F*(23,1) = 36.54, *p* < 0.001, *n*^2^ = 0.614] was also significant. The factor difficulty interacted with the distance [*F*(23,1) = 21.77, *p* < 0.001, *n*^2^ = 0.486]. Exploring the interaction of difficulty with distance revealed the interaction was mainly caused by a significant speed up in the *Difficult*-*Near* condition compared to the *Easy*-*Near* condition. In order to test these effects, we conducted paired sample *t* tests for two pairs of RTs grouped by distance. Two comparisions were made: RT *Near*-*Easy* (*M* = 0.388, SD = 0.056) versus RT *Near*-*Difficult* (*M* = 0.359, SD = 0.033) and RT *Far*-*Easy* (*M* = 0.430, SD = 0.075) versus RT *Far*-*Difficult* (*M* = 0.437, SD = 0.064). Only the RTs in the *Near* conditions were significantly different from each other [*t*(24) = 3.416, *p* = 0.002]. That is, RT in the *Near-Difficult* condition was significantly faster than the RT in the *Near-Easy* condition.

Additionally, there was a three-way interaction between difficulty, distance and group [*F*(23,1) = 4.919, *p* = 0.037, *n*^2^ = 0.176]. The three-way interaction was brought about by the relative differences in the amount of speed up across conditions for the two groups. This interaction was not explored further.

Overall, the RT data indicate that VGPs were faster in responding to the ball. All participants irrespective of difficulty or group responded faster in the *Near* conditions than the *Far* condition. Lastly, all participants responded the fastest in *the Difficult*-*Near* condition.

### Questionnaire

Subjects at the end of the experiment filled in a questionnaire that asked how *Difficult* they felt the condition where multiple presses were required to stop the ball (*Difficult*) was compared to the condition where one button press was required to stop the ball (*Easy*). The mean scores of difficulty were 2.85 (SD = 2.18) for VGPs and 2.91 (SD = 1.44) for NVGPs. A Mann–Whitney *U* test revealed no significant differences between the groups (*U* = 75.5, *p* = 0.88).

## Discussion

The aim of this study was to test whether objects are judged as being closer in conditions where it was more *Difficult* to stop an approaching object (*momentary* action capability). We also tested to see whether groups with contrasting inherent action capabilities (high vs. low *inherent* action capability) estimated distances of objects differently. The main results of this study are that, in the condition where it was *Easy* to stop the approaching ball, both VGPs and NVGPs were able to estimate the location of the approaching object accurately. However, when the object was more *Difficult* to stop, VGPs and NVGPs behaved differently. More specifically, NVGPs tended to estimate the approaching object as being closer than it actually was. The VGPs continued to be accurate in estimating the location of the approaching object. Additionally, the questionnaire measuring the relative difficulty in stopping the ball in the *Difficult* condition compared to the *Easy* condition indicated that both groups regarded the task to be reasonably *Difficult* (VGPs scoring a mean of 2.85 and NVGPs scoring a mean of 2.91).

The VGPs were always faster than NVGPs in responding to the colour change in the ball. Studies (Bialystok 2006; Dye et al. 2009) have shown that video game players show enhanced abilities at a range of cognitive- and motor-related tasks. The faster reaction times for VGPs found in our study are in line with these studies. Although the VGPs were faster, all subjects had equally high success rates with stopping the ball in both *Easy* and *Difficult* conditions. This was not surprising as the task was designed to ensure that subjects would be successful at stopping the ball. Subjects typically failed in the first few trials of the practice sessions in the *Difficult* block, but adapted quickly. These results indicate that although both groups found the task to be equally *Difficult* (as indexed by the questionnaire responses), VGPs were faster in stopping the ball.

The differences in location estimations between the NVGPs and VGPs in the *Difficult* block require some further interpretation. Many studies have demonstrated the presence of mechanisms that contribute to functional adjustments of visual information corresponding to moving objects. Firstly, due to transmission delay between information reaching the retina and travelling to the visual cortex, the visual cortex actively extrapolates the trajectory of moving objects to be able to keep up with the actual location (Berry et al. 1999). The neural basis of the motion extrapolation has been attributed to directionally selective neurons that directly activate other neurons that respond to the future locations of the moving stimulus (Fried et al. 2002). To be able to interact with a moving object, we have to compensate for the transmission delay between the motor cortex and the effectors, and also for the sluggishness of the responses of these effectors (Nijhawan et al. 2004). Therefore, motor commands are tailored to affect not the location where the object is when the command is issued, but at the location the object will be when the action needs to be performed. In order to accomplish this, the future locations of the object are computed and perhaps also stored as such, ahead in time (Nijhawan 1994).

The underestimations of location by the NVGPs in the *Difficult* condition might be seen as the result of an active compensation for the slower processing of detecting the stop signal and issuing the motor command to stop the moving ball. By perceiving the ball to be closer, NVGPs might have started the motor preparation ahead in time so that they would respond as quickly as possible when the stop signal was detected. VGPs, on the other hand, are trained in such situations and therefore might require less compensation. For instance, studies in persons who are proficient in certain motor skills such as expert musicians and athletes have shown that the overall cortical activation, required to perform a basic movement is lower than with controls (see Yang 2015 for a review). Also, in the *Easy* condition, such compensation would have been unnecessary for either group, as a small delay in responding to the stop signal would still have resulted in successfully stopping the ball.

The erroneous underestimations delivered by the NVGPs in our study may have been caused by forward shifts in the memory of the ball’s location, and not by the misperception of the location. Such an effect was coined *representational momentum* by Freyd and Finke (1984). Studies have shown that, when subjects were asked to report the final location of an object undergoing motion (e.g.: translational, rotational), they typically misremembered the object’s location further along its trajectory. Furthermore, subjects’ belief about the objects movement properties seemed to interfere with the effect. For example, when subjects were presented with objects that were associated with motion (such as rockets) and those were typically static (such as steeples), the former category showed greater forward shifts (Senior et al. 2000). Representational momentum accounts take into consideration the failure in stopping the dynamic updating of the object’s location in memory, interference caused by divided spatial attention (Hubbard and Ruppel 1999; Hayes and Freyd 2002) and the effect of action plans (Wexler and Klam 2001) on the final reported location.

In our study only the NVGPs underestimated locations, and only in the *Difficult* condition. One explanation is that VGPs and NVGPs used different strategies to perform the task. That is, VGPs could have been faster at stopping the dynamic updating of the ball’s location than NVGPs in the *Difficult* condition. Also, in the *Difficult* condition of our task, if the NVGPs monitored their critical response location (shifting their spatial attention completely to that location, or by dividing it in between the two locations) closely to be able to respond to the ball as soon as it reached that location, thereby dividing their attention between the moving ball and the critical location, it is possible that their dynamic memory representation of the ball was between the actual location and the critical location, yielding a forward displacement of the moving ball. Another possibility is that action capability is an independent factor that exerts its influence directly on location perception of dynamic objects.

A crucial difference between the embodied perception accounts and representational momentum accounts of object mislocalisation is that while the latter assumes that there are neural mechanisms and strategies that directly exert their influence on perception, the latter states that the forward shifts are caused at the representational level (memory shifts). Given our design, it is not possible to state whether our results were caused by the direct manipulation of perception (if the ball was viewed to be closer by), or by the manipulation at the level of perceptual judgements (if the ball was remembered to be closer by), or a combination of the two. We therefore offered alternative explanations that take both possibilities into account.

Although our study shows that VGPs were more accurate in estimating locations in all conditions, it is entirely possible that if the task difficulty was increased even further, they too would have started to underestimate locations. Therefore, the interaction between action capability and perception may lay along a continuum that is lower bounded by one’s inherent action capability. As long as the task is easier than what our inherent action capability allows us, perception of location may be unaffected by it. When the task becomes more *Difficult*, then perception may be altered to aid effective action. This possibility should be researched in future studies.

Lastly, we would like to compare the effects found in our study with those of Witt et al. (2008). An important difference between the two studies is that they compared the effects of task performance to perceptual judgements after the task was completed, while we manipulated skill level and difficulty while equalising the performance (success rates). Also, we asked for perceptual judgements during the task, on trials where no action to stop the ball was required. By using these two methods, we reduced the influence that emotional responses (negative or positive emotions caused by the subject’s performance) played on the perceptual judgements they reported. Therefore, the results found in our study may be viewed as an exportation of the results of Witt et al. (2008).

In conclusion, the results of our study show that both inherent and momentary action capabilities can interact to influence the location estimation. Mainly, our perceptual judgments of the location of an approaching object are adjusted dynamically in order to promote effective action execution. The location of the selfsame object, approaching in an identical fashion, but only separated by the expected difficulty in stopping it, could be judged differently.

## Footnotes

1 The analysis performed on the location estimates, found in the *Estimation* trials was repeated to the location estimates found in the *Response* trials. The raw location estimates for the near and far distance conditions, for each block (*Difficult* and *Easy*) and group (VGP and NVGP) into a paired sample *t* test. All tests yielded significant differences (*p* < 0.001, for all cases). A mixed between-group ANOVA was conducted with the same factors we used for the LEs from the Estimation trials. The results showed a main effect of difficulty [*F*(23,1) = 13.234, *p* = 0.001, *n*^2^ = 0.365] and distance [*F*(23,1) = 4.508, *p* = 0.045, *n*^2^ = 0.164]. The factor difficulty also interacted with group [*F*(23,1) = 13.886, *p* = 0.01 *n*^2^ = 0.376]. The factor group was also significant [*F*(23,1) = 24.512, *p* < 0.001, *n*^2^ = 0.516]. No other factors were significant. Pairwise comparisons between *Easy* and *Difficult* conditions revealed that locations were underestimated significantly for the *Difficult* condition (*p* = 0.001). We conducted one sample *t* test per difficulty and group to test which location estimation errors were significantly different from zero. The test revealed that only for the NVGP group were the LEs significantly different [*Easy*-Near: *M* = −5.12, SD = 3.28, *t*(11) = −2.480, *p* < 0.001, *Easy*-Far: *M* = −6.68, SD = 5.91, *t*(11) = −3.914, *p* = 0.002, *Difficult*-Near: *M* = −13.404, SD = 6.31, *t*(11) = −7.359, *p* < 0.001, *Difficult*-Far: *M* = −16.52, SD = 10.031, *t*(11) = −5.708, *p* < 0.001]. Thus, only NVGPs underestimated distances.
